# Communication Cues and Engagement Behavior: Identifying Advertisement Strategies to Attract Middle-Aged Adults to a Study of the Chronic Disease Self-Management Program

**DOI:** 10.5888/pcd17.190413

**Published:** 2020-06-25

**Authors:** Lindsey Horrell, George J. Knafl, Teresa Brady, Allison Lazard, Laura Linnan, Shawn Kneipp

**Affiliations:** 1 University of North Carolina at Chapel Hill, Gillings School of Global Public Health, Chapel Hill, North Carolina; 2 University of North Carolina at Chapel Hill, School of Nursing, Chapel Hill, North Carolina; 3 Clarity Consulting and Communications, Atlanta, Georgia; 4 University of North Carolina at Chapel Hill, Hussman School of Journalism and Media, Chapel Hill, North Carolina; 5 Lineberger Comprehensive Cancer Center, Chapel Hill, North Carolina

## Abstract

**Introduction:**

Low- and middle-income, middle-aged adults have high rates of disease and death from chronic disease, yet their participation in self-management programs is low. This may be because advertisements for such programs often target elderly, predominantly white, affluent adults. Our study used data from a parent randomized controlled trial to identify theoretically driven advertisement cues to engage low- and middle-income, middle-aged adults in the Chronic Disease Self-Management Program (CDSMP).

**Methods:**

A framework that combined the Elaboration Likelihood Model and Protection Motivation Theory was used to guide χ^2^ and regression analyses to assess relationships between advertisement cue preferences and 5 stages of cognitive engagement (cue processing, cognitive appraisal of the advertised study, motivation to enroll) and behavioral engagement of study participants (enrollment and program participation).

**Results:**

One advertisement cue (taking control of one’s future) and 1 cue combination (financial security and taking control of one’s future) were significantly associated with study enrollment, as were motivation to enroll and cue processing.

**Conclusion:**

These results can inform CDSMP recruitment efforts to better engage low- and middle-income, middle-aged adults in an effort to mitigate the disproportionate burden of chronic disease in this population.

**Summary**

**What is already known about this topic?**

The Chronic Disease Self-Management Program (CDSMP) has been linked to positive outcomes such as increased self-efficacy, decreased pain, and improved functioning. To date, CDSMP has predominantly engaged aging, affluent populations.

**What is added by this report?**

We identified advertisement cues and cognitive engagement constructs associated with enrollment of low- and middle-income, middle-aged adults in a study of the CDSMP.

**What are the implications for public health practice?**

By targeting CDSMP recruitment efforts to better engage low- and middle-income, middle-aged adults, we may begin mitigating the disproportionate burden of chronic disease in this population.

## Introduction

Although low- and middle-income, middle-aged adults have high rates of chronic disease, their participation in one of the most widely distributed and validated self-management programs, the Chronic Disease Self-Management Program (CDSMP), is low (1). Recent analyses of national samples of CDSMP participants showed that when adults aged 50 to 64 participated in the CDSMP, they were more likely to complete the program and to experience more positive health benefits than participants in other age groups (1–3). Engagement of this population may be low because advertisements for CDSMP overwhelmingly reflect the characteristics, needs, and concerns of aging, affluent, predominantly white adults (2,4–6). Extensive work in employee wellness initiatives demonstrated the importance of targeted recruitment strategies to engage middle-aged adults in health promotion programs (7–10); however, little has been done to identify recruitment strategies to effectively engage this population in CDSMP, which is typically offered outside the worksite (11).

**Figure 1:**
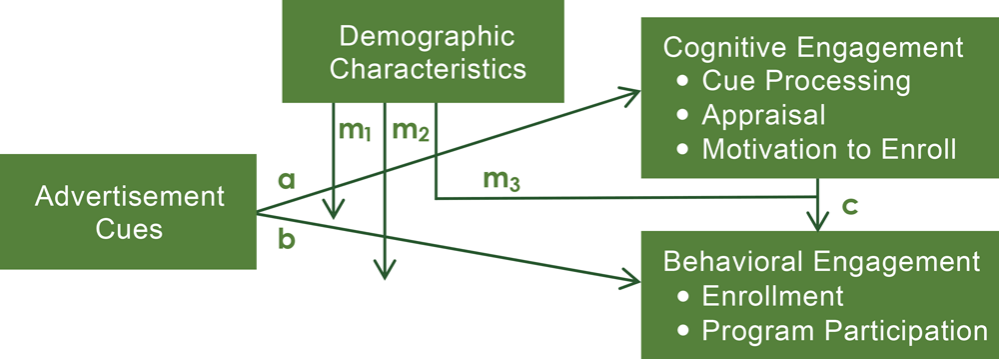
Advertisement engagement framework combines constructs from the Elaboration Likelihood Model and Protection Motivation Theory (12,13) to outline potential relationships among advertisement cues, cognitive engagement with study advertisements, and behavioral engagement outcomes, including potential moderating effects of demographic variables on the relationship between advertisement cues and cognitive engagement (m1) and behavioral engagement (m2), and the relationship between cognitive and behavioral engagement constructs (m3).

We conducted a theory-based evaluation of an advertisement used to engage low- and middle-income, middle-aged adults in a study of CDSMP that was guided by a theoretical framework combining constructs from the Protection Motivation Theory and Elaboration Likelihood Model (Figure 1). The objective of our research was to identify CDSMP advertisement cue preferences of low- and middle-income, middle-aged adults and their association with 3 cognitive engagement processes (cue processing, appraisal, motivation to enroll) and 2 behavioral engagement outcomes (enrollment and program participation) in a study of the CDSMP. Secondary objectives were to assess the association between cognitive and behavioral engagement in the parent study and to explore the moderating effect of demographic variables on each of these relationships. Results can be used to guide future efforts to engage this hardly reached population in CDSMP courses.

## Methods

### Participant recruitment

**Figure 2:**
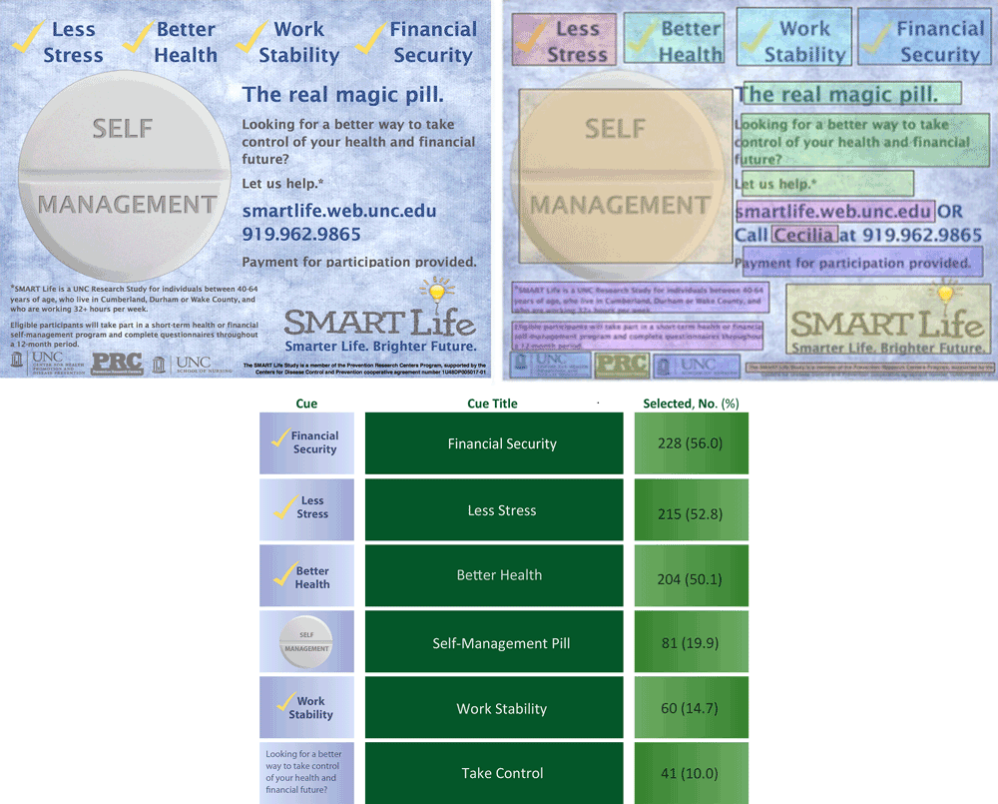
The most widely distributed SMART Life Study advertisement with a second version overlaid to indicate fields, defined a priori by study staff, for cue preference data collection. Beneath the image elements of the table that are cues are listed is a table describing responses to the cues contained in the advertisement.

Data for our study were collected through a parent randomized controlled trial, the SMART Life study, approved by the institutional review board at the University of North Carolina at Chapel Hill, which examined the health and economic effects of CDSMP among low- and middle-income, middle-aged adults. Participants were recruited for SMART Life through various approaches, including distributing 4 different print advertisements through pharmacies, asking employers to email the advertisement to employees, and visiting health fairs to distribute various printed advertisements in person. The analytic sample used in these analyses consisted of participants who viewed the most widely circulated SMART Life advertisement (Figure 2) and completed the online study registration survey. Adults were eligible to participate in SMART Life if they were 1) aged 40 to 64; 2) worked or resided in one of 5 study counties in North Carolina; 3) were employed at least 32 hours per week; 4) spoke, read, and wrote in English; 5) earned less than $60,000 annually; and 6) had been diagnosed with at least 1 chronic condition. The chronic condition was confirmed through self-report and defined as a condition that 1) had been diagnosed by a health care provider, 2) required ongoing attention and follow-up with a provider for 1 year or more, and 3) was likely to limit physical and social everyday activities at least some of the time. Participants indicated their diagnosis by selecting it from a list of 26 conditions or selecting “other” and writing in their diagnosis. For our study, data were collected through the SMART Life online registration and baseline surveys and the in-person CDSMP course rosters.

**Table:** 

Cue	Cue Title	Selected, No. (%)
Financial security	Financial security	228 (56.0)
Less stress	Better health	215 (52.8)
Better health	Less stress	204 (50.1)
Self-management	Self-management pill	81 (19.9)
Work stability	Work stability	60 (14.7)
Looking for a better way to take control of your health and financial future?	Take control	41 (10.0)

### Measures

**Cue preference.** Each participant was shown a picture of the study advertisement and asked to click on the 3 aspects of the advertisement that motivated them to begin the registration process. Each respondent’s selected cue preferences were captured through fields that were overlaid on the advertisement and remained invisible to participants until clicked (Figure 2). Data on whether or not a field was clicked were captured as a dichotomous variable and used in analyses.

**Engagement outcomes.** Engagement outcomes were selected according to a combination of the Elaboration Likelihood Model (ELM) and Protection Motivation Theory (PMT) (Figure 1). This combined framework outlines possible relationships between advertisement cues, 3 stages of cognitive engagement (cue processing, appraisal, motivation to enroll), and 2 stages of behavioral engagement (enrollment and program participation). Data on cue processing — in this study, the extent to which one feels involved with and finds the advertised material relevant — were captured by using the 10-item Personal Involvement Inventory, asking participants to rate the advertised material on factors such as importance, appeal, and relevance (14). Personal Involvement Inventory scores range from 10 to 70, and a higher score reflects a stronger involvement with the advertisement. The reliability of this scale was demonstrated in the study sample (Cronbach α of .85).

**Appraisal.** Data on a respondent’s assessment of both the advertised study and their chronic condition were captured by using the sum of 5 Likert-scale items. These items measured each participant’s perceived vulnerability to future health complications, perceived severity of health conditions, self-efficacy to participate in the study, response efficacy of the study, and cost of participating in the study. Appraisal scores ranged from 5 to 35, and reliability of the scale was demonstrated in the study sample (Cronbach α of .70). Motivation to enroll was measured by using a single item measured on a 7-point Likert scale asking participants to rate the extent of their intention to enroll in the study (lower scores signaled higher levels of motivation). Enrollment was defined as whether one continued past the registration survey to study eligibility questions. Program participation was measured among those assigned to the CDSMP and defined as one’s attendance at the first class of the 6-week course.

### Analyses

A series of linear and logistic regression analyses and χ^2^ analyses were conducted to identify cues and cue combinations that significantly correlated with each level of cognitive and behavioral engagement (cue processing, appraisal, motivation to enroll, enrollment, and program participation). First, the project team identified cues to include in regression analyses. Only cues selected by 5% or more of the sample were considered, to avoid sparse predictors with the potential for producing computationally degenerate regression models. Bivariate models were then developed to identify which cue preferences were significantly associated with engagement outcomes at *P* < .05. An SAS macro (SAS Institute, Inc) was used to adaptively search through interactions to identify effective singleton choices and 2-way and 3-way interactions for predicting engagement outcomes (15). We then conducted adaptive logistic regression analyses to assess the extent to which the cognitive engagement constructs were associated with behavioral engagement outcomes. Given the 3 measures of cognitive engagement, the adaptive search considered models individually on the basis of each construct, pairs of constructs, and all 3 together. Finally, each significant relationship was assessed for possible moderation by select demographic variables (sex, race, ethnicity, and annual household income) by using standard regression models. Moderation held if the interaction was significant (*P* < .05).

We also ran adaptive subanalyses to identify effective dichotomizations of both motivation to enroll and cue processing to assess the associations between dichotomized measures of these variables and subsequent behavioral engagement outcomes. Cue processing was defined as high if the Personal Involvement Inventory score was ≥58 and low otherwise; motivation was dichotomized as high if motivation equaled 1, and low otherwise. These cut points generated the best cross-validation scores used to compare models in adaptive analyses. Appraisal was not dichotomized, because it was significantly associated with enrollment as a continuous variable; however, because ours is the first study in which the items appraised were used and combined in the manner we used in our research, the team ran post-hoc analyses to determine whether the individual items used in the appraisal scale were associated with behavioral engagement outcomes.

## Results

Our analytic sample consisted of 407 adults who registered their interest in the SMART Life study after being exposed to the study’s most widely distributed advertisement (Figure 1). The mean age of the group was 48.8 (range, 26–66; standard deviation [SD], 8.3), and 79.3% of participants reported annual household incomes over $40,000. Most of the sample was female (81.1%), non-Hispanic (96.1%), and white (54.5%) (Table 1). Because of the participants’ progression through the study registration, eligibility, enrollment surveys, and expected attrition across these processes, the analytic sample used in each regression analysis differed. Program participation rates in the SMART Life study were low, limiting findings from analyses that included program participation as a behavioral outcome. Of the 407 participants included in our sample, 107 completed enrollment in the SMART Life study, and 53 were assigned to participate in CDSMP. Of the 53 assigned to CDSMP, only 8 completed the full course.

**Table 1:** Demographic Characteristics of Respondents (N = 407) to Most Widely Distributed SMART Life Study Advertisement^a^, 2015–2017

Characteristic	Accessed Registration n = 407	Motivation^b^, n = 343	Cue Processing^c^, n = 320	Appraisal^d^, n = 183	Enrolled in SMART Life, n = 107	Assigned to CDSMP, n = 53
**Sex**						
Female	334 (81.1)	282 (82.2)	261 (81.6)	150 (82.0)	93 (86.9)	49 (92.5)
Male	73 (17.9)	61 (17.8)	59 (18.4)	33 (18.0)	14 (13.1)	4 (7.6)
**Ethnicity**						
Hispanic	16 (3.9)	15 (4.3)	14 (4.4)	6 (3.3)	1 (1)	0 (0)
Non-Hispanic	391 (96.1)	328 (95.6)	306 (95.6)	177 (96.7)	106 (99.0)	53 (100)
**Race**						
Black	158 (38.8)	125 (36.4)	115 (35.9)	72 (39.3)	40 (37.4)	23 (43.4)
White	222 (54.5)	195 (56.9)	183 (57.2)	100 (54.6)	61 (57.0)	30 (56.6)
Other race	27 (6.6)	23 (6.7)	22 (6.9)	11 (6.0)	6 (5.6)	0 (0)
**Annual household income, $**						
≤39,999	84 (20.6)	59 (17.2)	57 (17.8)	42 (23.0)	26 (24.3)	13 (24.5)
40,000–79,999	191 (46.9)	169 (49.3)	157 (49.1)	90 (49.2)	54 (50.5)	25 (47.2)
≥80,000	132 (32.4)	115 (33.5)	106 (33.1)	51 (27.9)	27 (25.2)	15 (28.3)

a SMART Life advertisement (Figure 2). Values are number (percentage).

b Defined as the extent to which one intends to join the SMART Life Study; 343 participants completed this item.

c Defined as the extent to which one feels involved with and finds advertised material relevant; 320 participants completed this item questionnaire.

d Defined as one’s cumulative assessment of the advertised program and his or her chronic condition; 183 participants completed this item questionnaire.

### Cue preferences

The most widely selected cue was financial security (56.0%), followed by better health (52.8%), less stress (50.1%), self-management (shown on a pill as the main visual of the advertisement (Figure 2) (19.9%), work stability (14.7%), and take control, which asks the question, “Looking for a better way to take control of your health and financial future?” (10.0%). None of the cues was significantly associated with cognitive engagement outcomes, though a significant association was identified between selection of the take control cue and subsequent enrollment (χ^2^_1_ = 7.30; *P* = .007). Choosing both take control and financial security was also significantly associated with enrollment (OR: 4.08; 95% CI, 1.67–9.99; *P* = .002).

**Cognitive and behavioral engagement. **Appraisal was significantly associated with enrollment when measured as a continuous predictor (OR: 0.85; 95% CI, 0.79–0.92; *P* .001). When dichotomized, high motivation to enroll (1 vs >1; OR: 1.88; 95% CI, 1.17–3.03; *P* = . 007), was significantly associated with enrollment (Table 2), and participants with high cue processing had more than twice the odds of enrollment as those with low cue processing (≥58 vs <58; OR: 2.29; 95% CI, 1.16–4.49; *P* = .02). Among the 5 individual items that make up the appraisal scale, high perceived severity (OR: 0.45; 95% CI, 0.35–0.59; *P* .001) and perceived vulnerability (OR: 0.75; 95% CI, 0.63–0.90; *P* = .002) were significantly associated with lower odds of enrollment. No cognitive engagement measures were significantly associated with CDSMP participation.

**Table 2:** Bivariate Associations Between Cue Preference, Cognitive Engagement, and Behavioral Engagement Among Respondents to Most Widely Distributed SMART Life Study Advertisement^a^, 2015–2017

Dependent Variable	Path A, *F*_1_ (*P* Value)^b^			Path B, χ^2^ (*P *Value)^c^		Path C, OR (95% CI)^d^	
	Cue Processing, n = 320	Appraisal, n = 183	Motivation to Enroll, n = 343	Enrollment, n = 407	Program Participation^e^, n = 53	Enrollment, n = 407	Program Participation, n = 53
**Advertisement cues**							
Financial security	−0.87 (.22)	0.22 (.79)	−0.30 (.16)	0.85 (.36)	1.09 (.38)	—	—
Better health	1.20 (.09)	0.12 (.88)	0.07 (.75)	0.62 (.43)	2.71 (.14)	—	—
Less stress	−0.45 (.52)	0.44 (.59)	−0.13 (.53)	1.09 (.27)	0.04 (>.99)	—	—
Work stability	1.64 (.09)	0.79 (.44)	−0.15 (.60)	0.15 (.70)	0.22 (>.99)	—	—
Self-management pill	0.60 (.49)	1.63 (.10)	−0.10 (.71)	0.01 (.93)	0.01 (>.99)	—	—
Take control	0.44 (.69)	0.15 (.90)	−0.19 (.58)	7.30^f^ (.01)	0.74 (.65)	—	—
**High cue processing^g^**	—	—	—	—	—	2.29^h ^(1.16–4.49)	1.28 (0.24–6.70)
**Appraisal^i^**	—	—	—	—	—	0.85^f^ (0.79–0.92)	1.13 (0.96–1.33)
**High motivation to enroll^j^**	—	—	—	—	—	1.88 (1.17–3.03)^h^	0.81 (0.24–2.77)

Abbreviations: —, not applicable, CI, confidence interval; OR, odds ratio.

a SMART Life advertisement (Figure 2).

b Path A represents the relationship between advertisement cues and cognitive engagement outcomes (Figure 1).

c Path B represents the relationship between advertisement cues and behavioral engagement outcomes (Figure 1).

d Path C represents the relationship between cognitive and behavioral engagement outcomes (Figure 1).

e Fisher exact test run for cell counts <5.

f *P* ≤ .05.

g High cue processing was defined as ≥58 on the Personal Involvement Inventory, compared with <58.

h *P* ≤ .05 and predictor variables dichotomized.

i Defined as one’s cumulative assessment of the advertised program and his or her chronic condition.

j High motivation was defined as 1 on the single-item motivation Likert scale, compared to >1.

### Moderation

Although sex had a significant moderation effect within the relationship between choosing the take control cue and enrollment (χ^2^_1_ = 6.12, *P* = .013) and choosing both the take control and financial security cues and subsequent motivation (*F*_1_ = 3.93, *P* = .048), only 6 men in the analytic sample chose the take control cue, and only 1 chose both take control and financial security. No other demographic variable significantly moderated the associations explored in our research.

## Discussion

The objective of our study was to identify CDSMP advertisement cue preferences among low-and middle-income, middle-aged adults and better understand how advertisement preferences, appraisal and processing, and subsequent motivations relate to this population’s enrollment and participation in CDSMP research. Our results could be used as foundational data that researchers and the CDSMP program staff could build on to better engage this high-need, at-risk population in future CDSMP trials and community offerings. Our results indicate that marketing the potential financial benefits of enhanced self-management along with current messages of enhanced health may be an effective strategy to elicit the initial interest of the target population in CDSMP. However, additional research is needed to identify the financial benefits and barriers that may be preventing this population from participating in the program.

The top cue preferences all related to enhancing financial security, improving health, and reducing stress. These results may reflect the developmental stage of study participants, given that daily stressors tend to peak in middle adulthood and often relate to work, financial, and familial concerns (16–18). Implementing such cues aligns with previous research that states that gain-framed messaging — messaging that highlights the potential benefits of a behavior — is typically more effective at engaging individuals in prevention behaviors than a loss-framed approach, which would feature the consequences of not participating in the behavior (19).

One cue preference and one combination of cue preferences were significantly associated with engagement outcomes. Both selections of the take control cue alone and in combination with the financial security cue were positively associated with enrollment, suggesting participants may have been as motivated to enroll for financial reasons as for health reasons. Marketing the potential financial benefits of CDSMP (ie, the relationships among health, physical functioning, and productivity) and the control CDSMP could give participants over both their health and finances may be an effective strategy to effectively engage this population. Furthermore, the CDSMP staff could consider adding a course lesson or interactive activity on how to manage work and finances while dealing with a chronic disease in an effort to make CDSMP more marketable to low- and middle-income, middle-aged adults.

Regarding the association of cognitive engagement with behavioral outcomes, we found both motivation to enroll and appraisal to be significantly associated with enrollment. On further exploratory analyses, we found that cue processing and motivation were also associated with enrollment when dichotomized. Previous ELM literature suggests that increased personal involvement (or relevance) is associated with increased processing of the advertised material, suggesting that those in this study who processed the advertisement cues the most thoughtfully had subsequently higher odds of enrollment (12). Thus, research should continue to identify ways to market CDSMP in a way that increases the relevance of the program to low- and middle-income, middle-aged adults to increase their enrollment in the program. Our research suggests that including messages that allude to increased control over one’s finances and health may provide this enhanced program relevance.

According to PMT, people who perceive higher levels of severity, vulnerability, self-efficacy, and response efficacy and lower response costs should be more likely to enroll (13,20). In our analysis, however, those who reported high appraisal scores had significantly lower odds of enrolling. Each of the appraisal variables was also individually associated with low odds of enrollment, with this association being significant for perceived vulnerability and perceived severity. Many factors could explain why the appraisal construct did not perform as hypothesized. Given the stressors that characterize this population’s everyday life — such as meeting high work demands and caring for children and aging parents (16–18) — confounding variables related to time and availability could have prevented participants from enrolling in the study, regardless of how they appraised the program. This possibility is supported by the fact that few in the full SMART Life study sample who were assigned to CDSMP completed the full course (defined as attending ≥4 of the 6 class sessions) (21,22).

No constructs were identified that could significantly predict program participation, which could indicate that the proposed ELM+PMT framework adequately outlines relationships between advertisement cue exposure, cognitive engagement, and short-term behavioral engagement (enrollment). However, additional mediating variables should be added to the model when assessing long-term behavioral engagement (program participation) among low- and middle-income, middle-aged people. Thus, the proposed framework may be enhanced by incorporating variables that reflect barriers to participation as mediators of the currently outlined relationships. Previous barriers to recruitment have included lack of transportation, time, interest, or awareness of the program and a perception that people already manage their health well (23,24). When offering this course in the future, CDSMP leaders could consider evaluating ways to streamline the 6-week course to decrease time demands and increase uptake. Furthermore, program leaders may evaluate different modes of program delivery, such as courses offered online, via Skype, or in the workplace to decrease transportation barriers (related to time, cost, and availability) that may be hindering behavioral engagement or program participation of this at-risk population. Given the extensive work that has been done to identify strategies to engage employees in workplace wellness programs since the advent of the Affordable Care Act and the Prevention and Public Health Fund (7,8,10), incorporating CDSMP into workplace wellness initiatives may be an ideal venue to test future CDSMP engagement efforts among low- and middle-income, middle-aged adults while using lessons learned in our study regarding message preferences of this target population.

Finally, future research should be conducted with larger, more diverse samples to examine the moderating effects of sociodemographic variables on the relationships outlined in our research. The broader finding that demographic variables generally did not affect the studied relationships suggests that advertisement cues in place are engaging participants similarly across demographic factors. However, the fact that the analytic sample was predominantly white, non-Hispanic, and female suggests that current advertisement distribution strategies may not be reaching diverse audiences. Thus, additional research is needed to ensure CDSMP recruitment advertisements are disseminated through channels that effectively reach both male and racial/ethnic minority populations. These findings mirror other researchers’ calls for more investigation into how to better engage workers of low socioeconomic status in health promotion programs (25).

Our study had limitations. First, additional research is needed to assess the validity and reliability of the measures we used to capture data on the appraisal construct. Although each of these measures was adapted from those previously implemented in PMT literature and single item measures are routinely used in capturing cognitive appraisal constructs (26,27), our measures may not have captured every dimension of each appraisal component (perceived vulnerability, perceived severity, self-efficacy, response efficacy, response costs) (27–28). These variables are likely multidimensional and may not be assessed adequately using single item measures (29). Although scale development was outside the scope of our study, future research should focus on developing a valid and reliable scale of appraisal that can be used to further assess the relationships outlined in the proposed framework. Although the PII scores ranged from 10 to 70, each item was measured on a 3-point scale rather than the traditional 7-point scale, and scores were adjusted to match the traditional scoring. The resulting scale demonstrated reliability in this sample (Cronbach α of .85). Our analyses considered only cues selected by at least 5%, or 21, of the sample of 407 participants. Excluded cues might be of importance, but larger samples may be needed to make such assessments. Finally, only 53 participants in our analytic sample were assigned to the CDSMP. Thus, the study may not have been powered to identify relationships, particularly in terms of program participation, and the small sample may have affected the performance of the appraisal scale. Our study focused on an analytic subsample of the SMART Life study, and future research could reassess the relationships outlined in the proposed framework on the full SMART Life sample by categorizing cues seen on study advertisements not included in our analysis.

Given that members of our study population appeared to be motivated by both financial and health-related cues, CDSMP planners could consider better marketing of the potential financial benefits of enhancing self-management through CDSMP to increase engagement. Additional research is needed to assess the validity of measures available to study the constructs outlined in the proposed ELM+PMT framework and to identify environmental and contextual variables that may prevent people from participating in the CDSMP despite cognitive engagement. Although many people expressed interest in joining a study of CDMSP, efforts to deliver the program at a time and location and in a format that accommodates the hectic life events of middle adulthood might increase program participation. If CDSMP leaders do this and implement advertisements that convey messages of better health, financial security, and control over one’s future, they may begin to effectively engage low- and middle-income, middle-aged adults in CDSMP and thereby mitigate the disproportionately high burden of chronic disease in this population.
